# The Impact of Multidisciplinary Team Meetings on Patient Management in Oncologic Thoracic Surgery: A Single-Center Experience

**DOI:** 10.3390/cancers13020228

**Published:** 2021-01-10

**Authors:** Francesco Petrella, Davide Radice, Juliana Guarize, Gaia Piperno, Cristiano Rampinelli, Filippo de Marinis, Lorenzo Spaggiari

**Affiliations:** 1 Department of Thoracic Surgery, IRCCS European Institute of Oncology, 20141 Milan, Italy; juliana.guarize@ieo.it (J.G.); or lorenzo.spaggiari@unimi.it (L.S.); 2 Department of Oncology and Hemato-Oncology, Università degli Studi di Milano, 20141 Milan, Italy; 3 Department of Biostatistcs, IRCCS European Institute of Oncology, 20141 Milan, Italy; davide.radice@ieo.it; 4 Department of Radiotherapy, IRCCS European Institute of Oncology, 20141 Milan, Italy; gaia.piperno@ieo.it; 5 Department of Radiology, IRCCS European Institute of Oncology, 20141 Milan, Italy; cristiano.rampinelli@ieo.it; 6 Department of Thoracic Oncology, IRCCS European Institute of Oncology, 20141 Milan, Italy; filippo.demarinis@ieo.it

**Keywords:** multidisciplinary team meeting, thoracic oncology, tumor boards

## Abstract

**Simple Summary:**

Although the role of multidisciplinary team meetings (MDT) in thoracic oncology is well established, its real impact on decisional process is not well known yet. The aim of this paper is to quantify the MDT impact on the decisional clinical pathway, assessing the modification rate of the initial out-patient evaluation. Our results show a mean modification rate of 10.6%; the clinical settings “solitary pulmonary nodule” and “proven or suspected recurrence” disclosed higher modification rates (14.6% and 13.3%, respectively). When histology is available at out-patient evaluation, “pulmonary carcinoid” is the group with the lowest modification rate when compared to other histologies. In the light of our results, we suggest multidisciplinary discussion even in departments where MDT is not always routinely performed. Moreover, when discussing clinical perspectives with patients belonging to groups with a higher modification rate, physicians should emphasize the possible decisional variability in order to prevent patients’ disorientation or controversies.

**Abstract:**

Background: the aim of this paper is to quantify multidisciplinary team meeting (MDT) impact on the decisional clinical pathway of thoracic cancer patients, assessing the modification rate of the initial out-patient evaluation. Methods: the impact of MDT was classified as follows: confirmation: same conclusions as out-patient hypothesis; modification: change of out-patient hypothesis; implementation: definition of a clear clinical track/conclusion for patients that did not receive any clinical hypothesis; further exams required: the findings that emerged in the MDT meeting require further exams. Results: one thousand consecutive patients evaluated at MDT meetings were enrolled. Clinical settings of patients were: early stage lung cancer (17.4%); locally advanced lung cancer (27.4%); stage IV lung cancer (9.8%); mesothelioma (1%); metastases to the lung from other primary tumors (4%); histologically proven or suspected recurrence from previous lung cancer (15%); solitary pulmonary nodule (19.2%); mediastinal tumors (3.4%); other settings (2.8%). Conclusions: MDT meetings impact patient management in oncologic thoracic surgery by modifying the out-patient clinical hypothesis in 10.6% of cases; the clinical settings with the highest decisional modification rates are “solitary pulmonary nodule” and “proven or suspected recurrence” with modification rates of 14.6% and 13.3%, respectively.

## 1. Introduction

Oncologic diseases are complex clinical conditions requiring interaction between several specialists—with different skills and expertise—to offer the patients the best treatment strategies on the basis of the best available evidence [1]. A multidisciplinary team (MDT) consists of specialists with different backgrounds, skills and clinical experience, working together to recommend the best clinical pathway both in the case of planned treatments or to establish the most appropriate follow-up program [2]. MDT meetings in oncology can also be defined as tumor boards (TB); they offer several clinical benefits for global care: overall survival improvement [3,4], receipt of therapy [5,6], optimizing of treatment plan compared with pre-MDT hypotheses [7,8], staging accuracy [9] and global adherence to guidelines and international evidence-based recommendations [10,11,12,13].The MDT meeting can be considered as a common platform to coordinate the delivery of care by merging different clinical expertise in a single setting and can therefore be defined as a regularly scheduled discussion of clinical cases with the participation of physicians from different specialties such as surgeons, oncologists, radiotherapists, pulmonologists, pathologists, anesthesiologists, nurse specialists and other specialists when needed [14]. MDT meetings are suggested by many lung cancer treatment guidelines [15,16,17] but their organization and management is quite heterogeneous, varying across countries, hospitals and departments. Although the role of MDTs in lung cancer is well established today, their real impact on the decisional process is still not well known. The aim of this paper is to quantify the MDT impact on the decisional clinical pathway of thoracic cancer patients, assessing the modification rate of the initial out-patient evaluation, focusing on patients with different clinical settings referred to a high-volume oncologic thoracic surgical division.

## 2. Materials and Methods

The MDT meeting of the Division of Thoracic Surgery of the European Institute of Oncology is held weekly; attendees routinely include medical oncologists with a wide background in thoracic oncology, radiotherapists, interventional pulmonologists, thoracic surgeons, radiologists, a nurse case-manager and trainee specialists; other physicians are specifically invited on the basis of individual clinical cases, in particular pathologists, in case of unclear diagnosis. The meeting is coordinated by a senior physician; clinical cases are reviewed and presented by trainee specialists and all imaging exams are available on a maxi-screen. Each patient previously received an out-patient evaluation and then underwent a dedicated clinical track during which he/she was submitted to all exams, tests and procedures—both for oncologic and functional assessment—required by the referring physician at the time of out-patient access. After careful discussion, a final report is drawn up, the decision is recorded by a case manager and the patient is informed in person or by telephone about the results, depending on logistics and the patient’s preferences.

In the present study, the impact of the MDT on the previous out-patient program was classified as follows: (A) confirmation: same conclusions as the out-patient hypothesis (e.g., surgical indication confirmed); (B) modification: change of out-patient hypothesis (e.g., switch from surgical indication to radiotherapy or different treatment); (C) implementation: definition of a clear clinical track/conclusion for patients that did not receive any clinical hypothesis at out-patient access because of the lack of required exams or requiring further investigations before a definitive clinical conclusion (e.g., patient with no CT/PET/functional assessment or histology available at out-patient evaluation); (D) further exams required: the findings that emerged in the MDT meeting require further exams for a final decision.

With regard to clinical presentation at out-patient evaluation, patients were classified in the following “clinical settings”: (1) early stage lung cancer (stage I and II); (2) locally advanced lung cancer (stage IIIA and IIIB); (3) stage IV lung cancer; (4) mesothelioma; (5) metastases to the lung from other primary tumors; (6) histologically proven or suspected recurrence from previous lung cancer; (7) solitary pulmonary nodule (SPN); (8) mediastinal tumors; (9) other settings.

Written informed consent to undergo the procedures and for the use of clinical and imaging data for scientific or educational purposes, or both, were obtained from all patients; a blank copy of the written informed consent is provided.

### Statistical Methods

Patients’ characteristics were summarized and tabulated either by counts and percent or mean, median, Standard Deviation (SD) and Interquartile Range (IQR) for categorical or continuous variables, respectively. The MDT percent changes for each level (confirmation, modification, implementation, further exams) were plotted according to the clinical setting alongside 95% Confidence Intervals (CIs) computed using the binomial exact method. For each clinical setting, the change of out-patient hypothesis (modification) entered a univariate logistic regression analysis as the event of interest against other MDT levels, using sex, availability of histology, age and the out-patient days to MDT evaluation as independent variables. Multivariable logistic regression was conducted using only those variables showing a significant association with the modification event at the univariate analysis. Results are presented as Odds Ratios with 95% CIs. Comparison of proportions for the categorical variables were tested using the chi-square test, continuous variables were tested using either the unpaired t-test or the two-sample Wilcoxon test. All tests were two-tailed and considered significant at the 5% level. All analyses were conducted using SAS 9.4 (Cary, NC, USA).

## 3. Results

One thousand consecutive patients evaluated in the MDT meetings of the Division of Thoracic Surgery in 2019 were enrolled. There were 590 (59%) male and 410 (41%) female patients; mean age was 67 years (standard deviation SD 11.1); mean time between out-patient evaluation and final MDT meeting decision was 33 days (interquartile range IQR 27.0–43.5).

Clinical settings of patients were: (1) early stage lung cancer (174 pts—17.4%); (2) locally advanced lung cancer (274 pts—27.4%); (3) stage IV lung cancer (98 pts—9.8%); (4) mesothelioma (10 pts—1%); (5) metastases to the lung from other primary tumors (40 pts—4%); (6) histologically proven or suspected recurrence from previous lung cancer (150 pts—15%); (7) solitary pulmonary nodule (SPN) (192 pts—19.2%); (8) mediastinal tumors (34 pts—3.4%); (9) other settings (28 pts—2.8%, including inflammatory/infective diseases, non-specific adenomegalies, isolated pleural lesions, neurogenic tumors, aspergillosis, chest wall tumors).

The overall impact of MDT discussion on the previous out-patient program was: confirmation (580 pts—58%); modification (106 patients—10.6%); implementation (234 pts—23.4%). Eighty patients (8%) required further exams at MDT: 18 patients (22.5%) required biopsy; 26 patients (32.5%) required biopsy plus further imaging exams; 10 patients (12.5%) required endoscopy (colonoscopy and/or esophago-gastroscopy); 18 patients (22.5%) required further imaging exams and 8 patients (10%) required specialist consultation (orthopedic, hepatologist, vascular/cardiac surgeon). After these additional exams, the out-patient hypothesis was confirmed in 38 patients (47.5%), modified in 24 patients (30%) and implemented in 18 patients (22.5%) (Table 1).

**Figure 1:**
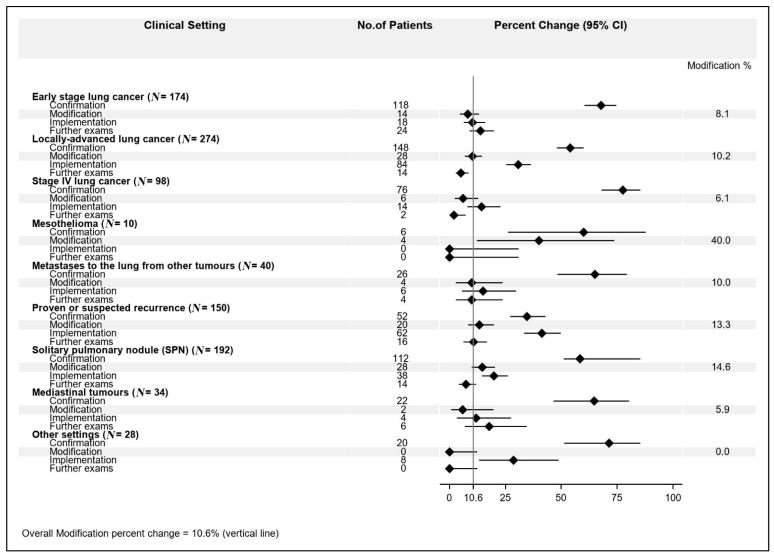
Multidisciplinary team meeting (MDT) impact percent changes according to clinical setting.

Among the different settings, we observed this MDT impact distribution: (1) early stage lung cancer: confirmation 67.8%; modification 8.1%; implementation 10.3%; further exams 13.8%; (2) locally advanced lung cancer: confirmation 54.1%; modification 10.2%; implementation 30.6%; further exams: 5.1%; (3) stage IV lung cancer: confirmation 77.6%; modification 6.1%; implementation 14.3%; further exams 2.0%; (4) mesothelioma: confirmation 60%; modification 40%; implementation 0%; further exams 0%; (5) metastases to the lung from other primary tumors: confirmation 65%; modification 10%; implementation 15%; further exams 4%; (6) histologically proven or suspected recurrence from previous lung cancer: confirmation 52%; modification 13.3%; implementation 41.3%; further exams 10.7%; (7) solitary pulmonary nodule (SPN): confirmation 58.3%; modification 14.6%; implementation 19.8%; further exams 7.3%; (8) mediastinal tumors: confirmation 64.7%; modification 5.9%; implementation 11.8%; further exams 17.6%; (9) other settings: confirmation 71.4%; modification 0%; implementation 28.6%; further exams 0% (Figure 1).

The highest modification rate (40.0%) was observed for the “mesothelioma” setting, though in only 4 patients out of 10 (95% CI: 12.1–73.8%), followed by “solitary pulmonary nodule” (14.6%, 95% CI: 9.9–20.4%) and “histologically proven or suspected recurrence from previous lung cancer” (13.3%, 95% CI: 8.3–19.8%). Next, the modification rates ranged from 10.2% (95% CI: 6.9–14.4%) for the “locally advanced lung cancer” going down to 0% (one-sided 97.5% CI: 0–16.8%) for “other setting”.

It is the case that 776 patients (77.6%) did not have a histologically proven diagnosis when they received their out-patient evaluation. On the contrary, 224 patients (22.4%) already had a histologic characterization when they received their out-patient evaluation: 128 patients (57.1%) suffered from lung adenocarcinoma; 40 patients (18%) from squamous carcinoma; 12 patients (5.4%) from pulmonary carcinoid; 6 patients (2.7%) from small cell lung cancer; 38 patients (17.0%) presented different histologic types. Among patients with available histologically proven diagnoses at out-patient evaluation, those affected by pulmonary carcinoid had a significantly lower modification rate (0%) when compared with patients with lung adenocarcinoma (12.5%), squamous cell carcinoma (15.0%), small cell carcinoma (33.3%) and other histologies (5.3%) (*p* = 0.03) (Table 2).

The settings “early stage lung cancer” and “locally advanced lung cancer” showed a significant modification rate association with the availability of histology at the out-patient evaluation. Specifically, the initial out-patient hypothesis was modified for all 14 (100%) early stage lung cancer patients whose histology was not available, compared to 98 (61.3%) patients at other MDT levels (*p* = 0.002), while for the locally advanced lung cancer patients, the out-patient hypothesis was changed only for 8 (28.6%) patients whose histology was not available compared to 152 (61.8%) for other MDT levels (*p* = 0.001) (Table 3).

Independent factors significantly associated with the modification event at the univariate analysis were the availability of histology vs. no availability (OR = 4.04, 95% CI: 1.71–4.05, *p* = 0.001) and the out-patient days to MDT evaluation (OR = 1.66, 95% CI: 1.15–2.41, *p* = 0.007), both for the locally advanced lung cancer setting and age (OR = 1.31, 95% CI: 1.03–1.68, *p* = 0.03) for the solitary pulmonary nodule setting (Table 4).

Multivariable analysis for the locally advanced lung cancer setting confirmed the significant association of both the availability of histology vs. no availability at out-patient evaluation (OR = 5.55, 95% CI: 2.23–13.7, *p* < 0.001) and days between out-patient evaluation and MDT discussion (OR = 1.04, 95% CI: 1.02–1.07, *p* < 0.001) (Table 5).

In this case, 222 patients (22.2%) did not receive any clinical hypothesis at out-patient access because of the lack of required exams.

## 4. Discussion

MDT meetings have been widely promoted to optimize the decision-making process for oncologic patients by improving coordination, communication and clinical discussion among physicians with different fields of expertise. However, the evidence that MDT meetings impact management was stronger than the evidence that they improve survival [3,18]. In a meta-analysis by Coory et coll., five studies on the impact of MDT on survival in lung cancer patients were found: among them, only two studies reported a modest 1-year survival increase in inoperable patients while the others did not disclose any advantage in terms of survival after the introduction of MDT meetings [19,20,21,22,23]. On the other hand, Pillay et coll.—in a systematic review on patient assessment, management and outcomes in oncology settings [6]—reported several studies among which the modification rate after MDT discussion ranged between 4% and 35% [24,25,26]. Moreover, very different scenarios have been observed in terms of MDT impact depending on each single specialty: for example, a modification rate of 27% was reported among gynecological patients after MDT discussion [27] and a modification rate of nearly 50% was reported among breast cancer patients after MDT meetings focusing on radiologic and pathologic data interpretation [28].

Our results show a global modification rate, after MDT discussion, of 10.6% which is consistent with the existing literature; moreover, the clinical settings “solitary pulmonary nodule” (SPN) and “proven or suspected recurrence” disclosed a higher modification rate of 14.6% and 13.3%, respectively. We may argue that the higher modification rate of these two settings may be due, on the one hand, to the intrinsic diagnostic challenge that both SPN and suspected recurrence represent by themselves; on the other hand, these are the clinical settings that most benefit from additional exams performed during diagnostic work-out, resulting in a more accurate diagnosis and clinical overview that may lead to modifying the out-patient initial decision.

The clinical setting “stage IV lung cancer” presents the lowest modification rate after MDT discussion (6.1%); this is probably due to the already clear clinical algorithm of a lung cancer metastatic patient at out-patient evaluation. In this setting, the surgical contribution is often limited to palliation of symptoms or diagnostic approach and can usually be well defined at out-patient approach; the clinical setting of “oligometastatic patients”—that may benefit from surgical treatment with curative intent [29,30]—was not considered in this paper.

Early stage lung cancer presents a very low modification rate after MDT of 8.1%; these patients are usually referred, at out-patient evaluation, for surgical therapy and a decisional switch at MDT is mainly due to cardio-pulmonary functional limitations, emerging during preoperative work-out, leading to radiotherapy as an alternative to surgical treatment [31,32]. Similarly, mediastinal tumors show a very low modification rate of 5.9%, due to clear surgical indications in the case of small and well-defined lesions as well as a clear multimodality treatment in the case of locally advanced tumors [33,34,35].

The vast majority of our patients (77.6%) had no histological diagnosis at out-patient evaluation, thus needing bronchoscopy or computed tomography(CT) -guided biopsy during clinical assessment before MDT discussion. Although—as expected—this further step conditioned a longer pre-MDT work-up compared with patients with available histology, the difference—albeit significant—was only 4 days (37 vs. 33, *p* < 0.0001).

In the group of patients with available histology at out-patient evaluation (22.4%), those with pulmonary carcinoid disclosed a significantly lower modification rate (0%) when compared to patients with lung adenocarcinoma (12.5%), squamous cell carcinoma (15.0%) small cell carcinoma (33.3%) and other histologies (5.3%) (*p* = 0.03). This was probably due to the fact that all patients in these groups (12 patients) belonged to early or locally advanced stages, thus the surgical indication formulated at out-patient evaluation was always confirmed during MDT discussion, as neither chemotherapy nor radiotherapy is indicated in this setting.

With regard to the “implementation” category, in this group we enrolled all patients (23.4%) for whom a clear clinical hypothesis was not possible, due to the lack of basic essential data provided at the time of out-patient evaluation. On the contrary, in the category “further exams required at MDT” (8.0%) we considered all patients that—despite receiving a clinical hypothesis and a complete diagnostic work-out—required additional new exams at the time of the MDT decision because of emerging unforeseen clinical conditions. In the vast majority of these latter cases, biopsies of new targets and further imaging exams were required, while in 10% of cases a specialist consultation was more rarely required to better study emerging vascular, cardiac, orthopedic and hepatic problems.

We have to point out some limitations of the study: as all of our patients are routinely presented and widely discussed at MDT meetings, we did not have any case-control group of patients not receiving MDT discussion, to search for an additional MDT value in terms of overall survival, disease-free survival or other clinical indicators, as reported in previous similar studies (Table 6) [36,37,38,39,40,41,42,43,44,45].

Moreover, because the population is quite heterogeneous with different tumors and stages, we could not compare disease outcomes of patients whose plan was changed with those without any change; we thus decided to focus on the decisional modification rate of MDT in order to identify the clinical settings that may most benefit from MDT discussion and for which we suggest clinical discussion even in departments where MDT is not always routinely performed. Moreover, when discussing clinical perspectives with patients belonging to clinical settings with higher modification rates, physicians should emphasize this aspect in order to prevent patient disorientation or controversies. Although this is a wide-population study, enrolling 1000 consecutive patients referred to a national high-volume referral cancer center, some clinical settings remain very infrequent, such as mesothelioma or other less frequent diseases, and so no clinical conclusion can be obtained for these groups of patients. Moreover, this paper is focused on the first-diagnosed cancer population and so some clinical scenarios of post treatment complications—such as post-resectional broncho-pleural fistula—are not evaluated [46].

## 5. Conclusions

MDT meetings impact on patient management in oncologic thoracic surgery by modifying the out-patient clinical hypothesis in 10.6% of cases, as similarly reported in other oncologic specialties. The clinical settings with the highest decisional modification rate are “solitary pulmonary nodule” and “proven or suspected recurrence” with modification rates of 14.6% and 13.3%, respectively. When histology is available at out-patient evaluation, “pulmonary carcinoid” is the group with the lowest modification rate when compared to other histologies. The modification rate in the settings “early stage lung cancer” and “locally advanced lung cancer” is significantly conditioned by the availability or not of histology at out-patient evaluation. When histology is not available at out-patient evaluation, the patients belonging to the “locally advanced lung cancer group” need more time (+4 days) to receive a definitive clinical decision.

In the light of our results, we suggest clinical discussion of these clinical settings even in departments where MDT is not always routinely performed; moreover, when discussing clinical perspectives with patients belonging to clinical settings with higher modification rates, physicians should emphasize this aspect in order to prevent patient disorientation or controversies.

## Figures and Tables

**Table 1:** Patients’ demography, clinical setting and MDT impact summary statistics, *N* = 1000.

Characteristic			Statistics ^a^
Age, years			65.2 (11.1)
Out-patient time/MDT (days)			33.0 (27.0–43.5)
Gender	Male		590 (59.0)
	Female		410 (41.0)
Histology	Available	not available	776 (77.6)
	Lung adenocarcinoma		128 (57.1)
	Squamous cell		40 (17.9)
	Pulmonary carcinoid		12 (5.4)
	Small cell lung cancer		6 (2.7)
	Other histology		38 (17.0)
	Overall available		224 (22.4)
Clinical settings	Locally advanced lung cancer		274 (27.4)
	Solitary pulmonary nodule		192 (19.2)
	Early stage lung cancer		174 (17.4)
	Proven or suspected recurrence		150 (15.0)
	Stage IV lung cancer		98 (9.8)
	Metastases to the lung from other tumors		40 (4.0)
	Mediastinal tumors		34(3.4)
	Mesothelioma		10 (1.0)
	Other		28 (2.8)
Impact of MDT discussion	Confirmation		580 (58.0)
	Modification		106 (10.6)
	Implementation		234 (23.4)
	Further exams		80 (8.0)
	Specific Examination	Biopsy	18 (22.5)
		Biopsy + further imaging exams	26 (32.5)
		Colonoscopy/esophago-gastroscopy	10 (12.5)
		Further imaging exams	18 (22.5)
		Specialist consultation ^b^	8 (10.0)

^a^ Statistics are: Mean (SD) for Age, Median (IQR) for Out-patient time/MDT days; SD = Standard Deviation, IQR = Interquartile Range, N (%) on available data for histology, N (%) otherwise; ^b^ orthopedic, vascular/cardiac surgeon, hepatologist.

**Table 2:** Frequency distribution of histology type by MDT impact, N = 224.

Histology	Overall *N* (%)	Confirmation *N* = 156	MDT Impact Level, N (Row %)		Further Exams *N* = 18	*p*-Value
			Modification *N* = 26	Implementation *N* = 24		
Lung adenocarcinoma	128 (57.1)	88 (68.8)	16 (12.5)	12 (9.4)	12 (9.4)	
Squamous cell	40 (18.0)	20 (50.0)	6 (15.0)	8 (20.0)	6 (15.0)	
Pulmonary carcinoid	12 (5.4)	12 (100)	0	0	0	0.03
Small cell lung cancer	6 (2.7)	4 (66.7)	2 (33.3)	0	0	
Other	38 (16.7)	32 (84.2)	2 (5.3)	4 (10.5)	0	

**Table 3:** Frequency distribution of the availability of histology at out-patient evaluation according to the clinical setting.

Clinical Setting	Histology	MDT Impact, N (col %)		*p*-Value
		Modification	Other	
Early Stage Lung Cancer	Not yet available	14 (100)	98 (61.3)	
	Available	0	62 (38.8)	0.002
Locally Advanced Lung Cancer	Not yet available	8 (28.6)	152 (61.8)	
	Available	20 (71.4)	94 (38.2)	0.001
Stage IV Lung Cancer	Not yet available	4 (66.7)	66 (71.7)	
	Available	2 (33.3)	26 (28.3)	1.00
Mesothelioma	Not yet available	2 (50.0)	4 (66.7)	
	Available	2 (50.0)	2 (33.3)	1.00
Metastases to the lung from other tumors	Not yet available	4 (100)	34 (94.4)	
	Available	0	2 (5.6)	1.00
Proven or suspected recurrence	Not yet available	20 (100)	120 (92.3)	
	Available	0	10 (7.7)	0.36
Solitary pulmonary nodule	Not yet available	28 (100)	164 (100)	
	Available	0	0	-
Mediastinal tumor	Not yet available	2 (100)	30 (93.8)	
	Available	0	2 (6.3)	1.00
Other	Not yet available	0	28 (100)	
	Available	0	0	-

**Table 4:** Univariate Analysis of factors associated with modification by clinical setting.

Clinical Setting	Factor	Level	OR (95% CI)	*p*-Value
Early Stage Lung Cancer*^a^*	Sex	Female	1	
		Male	1.21 (0.35–4.16)	0.76
	Age		0.95 (0.72–1.25)	0.71
	Out-patient time/MDT days		1.57 (1.00–2.47)	0.05
Locally Advanced Lung Cancer	Sex	Female	1	
		Male	1.08 (0.48–2.43)	0.76
	Histology	Not yet available	1	
		Available	4.04 (1.71–0.45)	0.001
	Age		1.21 (0.97–1.52)	0.10
	Out-patient time/MDT days		1.66 (1.15–2.41)	0.007
Stage IV Lung Cancer	Sex	Female	1	
		Male	1.17 (0.20–6.72)	0.86
	Histology	Not Available	1	
		Available	4.57 (0.79–26.4)	0.09
	Age		0.91 (0.61–1.36)	0.64
	Out-patient time/MDT days		0.50 (0.17–1.46)	0.20
Mesothelioma*^b^*	Histology	Not Available	1	
		Available	2.00 (0.15–26.7)	0.60
	Age		Not estimable	-
	Out-patient time/MDT days		2.56 (0.64–10.3)	0.18
Metastases to the lung from other tumors *^c^*	Sex	Female	1	
		Male	Not estimable*^d^*	0.76
	Age		0.59^§^ (0.23–1.51)	0.27
	Out-patient time/MDT days		0.96 (0.32–2.86)	0.94
Proven or suspected recurrence*^e^*	Sex	Female	1	
		Male	1.77 (0.68–4.63)	0.25
	Age		1.05 (0.84–1.32)	0.67
	Out-patient time/MDT days		0.66 (0.38–1.15)	0.94
Solitary pulmonary nodule*^f^*	Sex	Female	1	
		Male	0.99 (0.44–2.23)	0.99
	Age		1.31 (1.03–1.68)	0.03
	Out-patient time/MDT days		1.11 (0.74–1.66)	0.61
Mediastinal tumor*^g^*	Sex	Female	1	
		Male	Not estimable *^d^*	-
	Age		0.77 (0.42–1.42)	0.41
	Out-patient time/MDT days		0.99 (0.17–5.71)	0.99

***^a,c,e,f,g^*** Histology not available for all patients on Modification level; *^b^* Male patients only;*^d^* No change of out-patient hypothesis (modification) for all female patients; Odds Ratios for age and out-patient time/MDT days are associated with 5 years unit increase and 15 days delay unit increase respectively; OR = Odds Ratio; 95% CI = 95% Confidence Interval.

**Table 5:** Multivariable analysis of factors associated with modification for the locally advanced lung cancer setting.

Factor	Level	OR (95% CI)	*p*-Value
Histology	Not yet available	1	
	Available	5.55 (2.23–13.7)	< 0.001
Out-patient time/MDT days *^a^*		1.04 (1.02–1.07)	< 0.001

***^a^***Mean out-patients/MDT days 37 vs. 33 for histology not yet available vs. histology available, *p* < 0.001.

**Table 6:** The role of MDT meeting in cancer settings in recent literature.

Author (ref n.)	Year	Clinical Setting
Klarenbeek SE (36)	2020	Lung cancer
Shashi KK (37)	2020	Esophageal cancer
Crichi B (38)	2020	Venous thromboembolism in cancer patient
Graetz DE (39)	2020	Pediatric oncology
Karas PL (40)	2020	Medicolegal aspects in cancer care
Zhao S (41)	2020	Esophageal cancer
Dijkstra S (42)	2020	Pediatric oncology
Warner R (43)	2021	Urologic cancer
Liam CK (44)	2020	Lung cancer
Pluyter JR (45)	2020	Lung cancer

## Data Availability

Available on request.
